# Reactive Oxygen Species in Plants: From Source to Sink

**DOI:** 10.3390/antiox11020225

**Published:** 2022-01-25

**Authors:** Sheikh Mansoor, Owais Ali Wani, Jafar K. Lone, Sweeta Manhas, Navneet Kour, Pravej Alam, Ajaz Ahmad, Parvaiz Ahmad

**Affiliations:** 1 Division of Biochemistry, FBSc, SKUAST-J, Jammu 180009, India; mansoorshafi21@gmail.com (S.M.); sweetamanhas1@gmail.com (S.M.); kour.navneet13@gmail.com (N.K.); 2 Division of Soil Sciences and Agricultural Chemistry, SKUAST Kashmir, Jammu 193201, India; owaisaliwani@gmail.com; 3 ICAR-NBPGR, Division of Germplasm Evaluation Pusa Campus, New Delhi 110012, India; jaffar.kl12@gmail.com; 4 Department of Biology, College of Science and Humanities, Prince Sattam Bin Abdulaziz University, Alkharj 11942, Saudi Arabia; alamprez@gmail.com; 5 Department of Clinical Pharmacy, College of Pharmacy, King Saud University, Riyadh 11451, Saudi Arabia; ajukash@gmail.com; 6 Department of Botany, GDC, Pulwama 192301, India

**Keywords:** reactive oxygen species, production, stress, signaling, cell death

## Abstract

Reactive oxygen species (ROS, partial reduction or derivatives of free radicals) are highly reactive, dangerous and can cause oxidative cell death. In addition to their role as toxic by-products of aerobic metabolism, ROS play a role in the control and regulation of biological processes such as growth, the cell cycle, programmed cell death, hormone signaling, biotic and abiotic stress reactions and development. ROS always arise in plants as a by-product of several metabolic processes that are located in different cell compartments, or as a result of the inevitable escape of electrons to oxygen from the electron transport activities of chloroplasts, mitochondria and plasma membranes. These reactive species are formed in chloroplasts, mitochondria, plasma membranes, peroxisomes, apoplasts, the endoplasmic reticulum and cell walls. The action of many non-enzymatic and enzymatic antioxidants present in tissues is required for efficient scavenging of ROS generated during various environmental stressors. The current review provides an in-depth look at the fate of ROS in plants, a beneficial role in managing stress and other irregularities. The production sites are also explained with their negative effects. In addition, the biochemical properties and sources of ROS generation, capture systems, the influence of ROS on cell biochemistry and the crosstalk of ROS with other signaling molecules/pathways are discussed.

## 1. Introduction

Oxygen-evolving photosynthetic organisms introduced O_2_ to the reducing atmosphere of Earth about 2.7 million years ago. This was further accompanied by the production of reactive oxygen species (ROS) as by-products of various metabolic reactions. In plants, different cellular organelles such as chloroplasts, peroxisomes and mitochondria are confined for the generation of ROS. In addition to the above-mentioned sites of ROS production, cell walls and plasma membranes containing peroxidases and amine oxidases, respectively, lead to ROS production in case of environmental stress. ROS include singlet oxygen (^1^O_2_), hydrogen peroxide (H_2_O_2_), superoxide (O^2−^) and hydroxyl radicals (OH^.^). They exhibit a variety of physiological responses, structural changes and the degradation of macromolecules [1]. In the process of photosynthetic electron transport, chloroplasts consistently produce oxygen, which becomes eliminated by reduction and assimilation. In photosystem I (PS I) and the photorespiratory cycle, the photoreduction of O_2_ to a superoxide radical takes place by reduced components of the electron transport system. In the case of stress and the low availability of CO_2_, the enzyme RUBP (ribulose-1,5-bisphosphate) carboxylase/oxygenase prefers oxygen as a substrate to catalyze a reaction. It leads to glycolate production, which on oxidation, produces H_2_O_2_ in peroxisomes via glycolate oxidase. In the case of the plasma membrane, multimeric cytochromes present in the electron transport chain favor the reduction of oxygen to superoxide (O_2_^−^) via NADPH-dependent oxidases. Unlike the plasma membrane, pH-dependent peroxidase, oxalate oxidase and amine oxidase are responsible for ROS generation in the apoplast [2,3,4].

In mammals, mitochondria are major sites for ROS production, whereas a less-prominent role has been observed in plants [5,6,7,8]. Many studies assume that ROS production is a basic symptom of plant toxicity under abiotic stress. As the production of ROS increases, the phytotoxicity also rises. In such toxic conditions, plant growth and metabolism are adversely affected, thereby leading to less crop productivity. Despite the fact that ROS are involved in the cellular metabolism of plant cells, their overproduction under stress conditions causes photooxidative damage to various cellular entities such as carbohydrates, lipids, nucleic acid and proteins [9]. In addition to ROS generation, their regulation is mainly achieved by defensive mechanisms, including enzymatic as well as non-enzymatic antioxidants. In the enzymatic system, superoxide is responsible for the conversion of hydroxyl to hydrogen peroxide, which is catalyzed by peroxidase and catalase (CAT) to produce water and dioxygen. The non-enzymatic scavenging mechanism is mediated by low-molecular-mass antioxidants, namely glutathione, ascorbic acid, carotenoids, flavonoids, etc., by targeting hydroxyl radicals and singlet oxygen. In case of an imbalance between the production and scavenging of ROS, plant cells generate oxidative stress, eventually leading to oxidative modification and ultimately cell death [10]. As a secondary messenger, ROS take part in cell division, differentiation, biotic and abiotic responses as the cells are maintained in a reduced state due to low ROS levels [11]. Therefore, ROS play a crucial role, and maintaining a threshold level is important as it is necessary for plant growth and development. Apart from the damaging effect, ROS are also considered to be a signaling molecule playing an immense role in plant growth and development, stress-responsive genes and leading to programmed cell death. Through various redox reactions, the signal is transported to the nucleus, which goes downstream via the mitogen-activated protein kinase (MAPK) pathway. It helps in the development of enhanced tolerance in several cellular mechanisms under stress conditions [12,13]. Many studies demonstrated the critical role of H_2_O_2_ in several crop plants, such as rice, maize, wheat, bean and soybean, in the regulation of a stress-responsive environment. Apart from ROS, reactive nitrogen species, reactive sulfur species and reactive carbonyl species exhibit roles in signaling as well as in the stress tolerance mechanism [8].

Various stress factors, including drought, salinity, temperature, heavy metal, etc., are known for disturbing the equilibrium maintained between ROS generation and its scavenging. In such conditions, the capability of plant tolerance mainly relies upon some prime factors: extremity and time duration of stress; variation in growth; how much faster the plant is likely to adapt with respect to changes in the environment [14]. At times, manifold stressors are reported to occur simultaneously, which thereby leads to a rapid decline in crop productivity [2,14]. To keep up with drastic environmental conditions, plants have evolved various stress-responsive genes encoding their respective proteins required for activation as well as regulation of ROS. Herein, among several genes, transcription factors play a prominent role in providing increased tolerance, and many studies have demonstrated their role in the successful management of abiotic stress [15,16,17,18]. In this review, we provide detailed insight regarding ROS generation, their biochemistry and the signaling mechanism in plants to effectively cope with abiotic stress. In particular, we summarize strategies/plant disease resistance mechanisms, the antioxidant defense systems and also the crosstalk of ROS with the signaling molecules. 

## 2. ROS in Disease Resistance

The adaptability of plants towards pathogen attack and strategies of co-evolution have always been incremental and of prime importance for agricultural production systems over the years. Plant susceptibility and resistance against foreign invasion by pathogens are under the control of recognition and signaling pathways between the host and the pathogen. Microbe/pathogen-associated molecular patterns, i.e., MAMPs/PAMPs and Avr (avirulent) gene products that correspond to their host receptors (plants) along with R (resistance) genes, lead to the activation of a signaling cascade that generates ROS, phytoalexins and anti-microbial genes. This further activates a wide range of plant defense genes proficient against a wide range of pathogens. In plants, the electron transport chain in mitochondria and chloroplasts, as well as peroxisomal photorespiration, generate ROS [19]. RBOH (respiratory burst oxidase homolog) genes encoding NADPH oxidase, polyamine oxidase (PAO)-mediated degradation of spermidine and oxalate oxidase are involved in the production of apoplastic ROS in the plasma membrane. 

The most stable and abundant ROS in plants include H_2_O_2_ (hydrogen peroxide), superoxide (O_2_^−^), (hydroxyl) (OH) and singlet oxygen (^1^O_2_). There is a rapid interconversion between the four that provides a higher functional variability. Of all the four classes, H_2_O_2_ is highly stable and ROS-transported via aquaporin membranes [20]. Different concentrations of ROS generated have different physiological outcomes. In small concentrations, signaling functions are exerted by ROS. However, the extensive accumulation of ROS may lead to cell death caused by its damaging oxidative effects on nucleic acids, proteins and lipids. An interplay between calcium channels, NADPH oxidases (NOX) and calcium fluxes induced during oxidative stress generates a ROS wave, which can transduce long-distance signals [21]. An ROS wave is a respiratory burst-homolog-D (RBOHD)-mediated cell–cell self-propagating process of ROS production. Once triggered, it results in enhanced production of ROS in a single cell, which acts as a sensory signal for other cells to increase their ROS production. In a recently reported novel function of the ROS wave, there is coordination between different induced stresses and the whole-plant systemic stomatal response [21,22,23]. The ROS wave is a cell-to-cell auto-propagating process of ROS production mediated by the respiratory burst homolog D (RBOHD) protein [4,14,21,24]. Once triggered in a single cell, it causes the enhanced production of ROS by the cell. This results in the accumulation of ROS at the apoplast (RBOHD produces ROS at the apoplast) [25].

## 3. Regulation of MAP Kinase 

**Figure 1:**
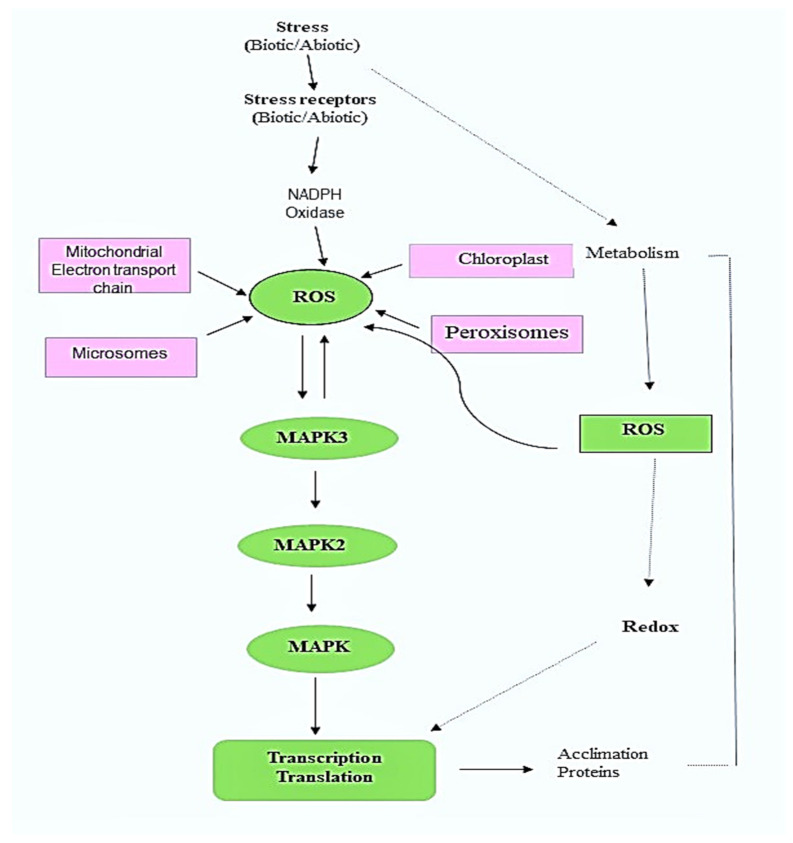
ROS signaling, the activation of the MAPK cascade and redox ROS homeostasis in the cell. In biotic and abiotic stressors, the reactive oxygen species (ROS) signaling pathway is regulated by mitogen-activated protein kinases (MAPK). ROS is a common messenger that is produced in response to both the stress response and the MAPK cascade. Despite having a similar MAPK signaling regulator, the plant’s reaction to both stressors is distinct.

Among the mitogen-activated protein kinases (MAPKs), MAP2Ks and MAP3Ks represent a class of key signal transduction proteins that are triggered in response to a plethora of developmental and environmental factors. ROS accumulation is well-known to be the activating factor of MAPK signaling under biotic and abiotic stress conditions. MAPK pathways are three-rung kinases of MAPKKK that phosphorylate and activate MAPKK, which in turn activate MAPKs after phosphorylation. The phosphorylation of MAPKs, once activated, leads to the inactivation or activation of various target proteins as well as transcription factors. MAPKS are involved in pathogen-mediated signal transduction (biotic) and abiotic stress such as drought, cold, salinity, wounding, ROS, ozone and hormone stimuli [26,27]. Although ROS production inevitably mediates stress-induced defense responses, it is equally important to limit over-accumulation of ROS. To prevent oxidative stress induced by ROS overproduction, the cell responds to the increased production of ROS by an antioxidant defense system, which has a key role in maintaining the levels of ROS. Thus, redox control of TFs (transcription factors) is critical in defining the cellular response to oxidative stress and gene expression profiling (Figure 1). Several genetic studies have reported the significance of antioxidant enzymes in maintaining a positive correlation between plant stress tolerance and the expression of enzymes responsible for ROS regulation. The scavenging of ROS is accomplished via non-enzymatic as well as enzymatic pathways. Low-molecular-weight metabolites glutathione, alpha-tocopherol, ascorbate, flavonoids and carotenoids mediate the non-enzymatic antioxidant pathway. 

The major enzyme classes involved in the enzymatic regulation of ROS include ascorbate peroxidases, superoxide dismutases, catalases, glutathione reductases, dehydroascorbate reductases and monodehydroascorbate reductases. Thio-, gluta- and peroxiredoxins, along with peroxidases and glutathione peroxidases, are potent ROS scavengers. Functional and sequence analyses of the Arabidopsis genome revealed 80 MAPKKKs, 10 MAPKKs and 20 MAPKs, presenting a similar gene catalog to those observed in other plant genomes. Along with MAPK proteins, plant hormones have a key role in acclimation responses to abiotic stress. Recent studies have elucidated an interplay between ROS and phytohormones during abiotic stress. An increase in ROS levels under abiotic stress not only affects the transcription of genes, metabolic flux and proteome, but also modulates the function and level of phytohormones [28]. A molecular link between oxidative stress and the auxin signal transduction pathway has long been reported [29,30,31,32,33,34]. The production of ROS during stress (abiotic) results in changes in auxin gradients that, in turn, reduce auxin-induced signaling. Auxins can also induce ROS production and help in maintaining ROS homeostasis, exhibiting an association between oxidative stress and auxin signaling [35]. Auxins also play a role in the activation of RAC/ROP (Rho-GTPase), which further interact with NADPH oxidases that cause apoplastic production of ROS. On the contrary, ROS regulate the transport of auxins via PIN gene regulation and the relocation of auxin exporters. Auxin signaling is followed by a mitogen-activated protein kinase (MAPK) cascade, resulting in crosstalk between auxins, MAPKs and ROS. It has been reported that MPK12, an Arabidopsis protein, interacts with IBR5 (indole-3-butyric acid response), a MAPK phosphatases specifically, which on dephosphorylation inactivates MPK12. Thus, MPK12 acts as an IBR5 phosphatases substrate that is activated by auxins in vivo, and the suppression of MPK12 results in auxin-responsive gene expression, suggesting the role of MPK12 as a negative regulator in Arabidopsis auxin signaling [8].

## 4. ROS Mediated Stress Responses—Stress, Hormone and ROS Crosstalk

ROS is a commonly produced factor under both abiotic as well as biotic stress. In Arabidopsis, mitogen-activated protein kinase kinase kinase 1 (MEKK1), MAP3K is activated under abiotic stress conditions such as wounds, cold, salt and drought, and in biotic stress responses against fungal and bacterial elicitors, MEKK1 is known to be activated with the production of ROS in such stimuli. When there are abiotic stimuli, the MKK2-MPK4/6 module is activated by MEKK (mitogen-activated protein kinase kinase 2) [36]. On the other hand, biotic stimuli result in the activation of the MKK4/5-MPK3/6-VIP1/ACS6 module. MEKK1-MKK1/2-MPK4 acting upstream of MKS1/WRKY33 also works in mediating pathogen-related responses. ROS, when produced under different kinds of environmental stresses, such as heavy metal, ozone, abscisic acid (ABA) treatment and biotic stress, activates MPK6 and MP3K, which further mediate different responses [36,37]. MPK6 and MP3K act downstream of Ser/Thr protein kinase OXI1 in Arabidopsis, which has two different biological roles; it stimulates fungal pathogen resistance in plants and stimulates root development. In rice, hydrogen peroxide activates MPK6 and MPK3 kinases, which are involved in resistance against abiotic stresses such as salt, UV rays, cold and heavy metal, as well as biotic stress resistance against fungal pathogen. What makes a single pathway act in two processes can be explained from the above-cited examples where crosstalk mediated by ROS among MAPKs suggest that in different environmental factors, the production of ROS results in the activation of MAPKs (similar), but their interaction and final response towards different stresses becomes fundamentally different. It seems that ROS acts as a messenger that is involved in encoding information for the activation of different responses [37]. The complexity of the intricate interplay between ROS and the plant hormones further increases under multiple stress conditions [38]. For instance, interactions between ROS, ABA, jasmonic acid (JA) and salicylic acid (SA) are associated with the regulation of stomatal movement during stress combination. It has been reported that abil (ABA-insensitive mutants), having impaired ABA function, and ABII (ROS regulated protein), were sensitive towards the combined stress of drought and high temperatures. The mutants were also sensitive to combined high temperature and salinity stresses, thus highlighting the role of ROS–ABA interactions in the acclimation of plants to combination stress. Following a combination of high temperatures and drought stress, the stomatal closure was accompanied by an increase in hydrogen peroxide and JA concentration with a decrease in SA concentration in the leaves, which suggests the possibility of a canonical role of H_2_O_2_ and JA in stomatal responses independent of ABA [39,40,41].

## 5. Defense System against ROS Production and Accumulation 

### Introduction to the Defense System against ROS Production and Accumulation 

**Figure 2:**
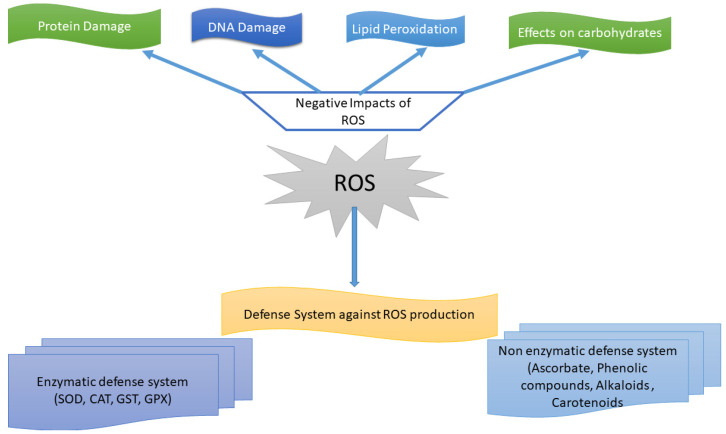
Effects of oxidative stresses on plant parts and different defense mechanisms. In plants, ROS cause serious damage to the cells by inhibiting proteins, DNA and other metabolic pathways. Conversely, the defense system is activated in the plants against ROS to regulate its functional activity by activating different enzymatic and non-enzymatic antioxidant agents.

The ROS defense system in plants and its various processes and components were discovered at the end of the twentieth century [34,42,43,44,45]. Recently, it was concluded that the defense system against ROS is not only due to the scavenging system, but it also constitutes an enzymatic as well as non-enzymatic defense system, and these defense systems are initiated by various external environmental stresses [46]. Routine cellular metabolism results in the production of ROS, which is regulated by various enzymatic and non-enzymatic defense systems (Table 1). Enzymatic defense systems include APX, CAT, SOD and GPX, whereas non-enzymatic systems include glutathione (GSH), ascorbic acid (AA), phenolic compounds and tocopherols (TOCs) [45,47,48]. Plants possess a multifarious ROS defense system comprised of both enzymatic as well as non-enzymatic system. ROS production, as well as scavenging, can be located in different cell components, such as peroxisomes, chloroplasts and mitochondria, and there is strong coordination between these organelles in the case of such pathways [49]. Under normal conditions, there is equilibrium between the production and scavenging of ROS in plants, but under stress, this equilibrium is disturbed, leading to an elevation in ROS levels [49], which leads to oxidative stress to cell components, whereas in more evolved plants, there is a natural defense system to counter this rise in ROS levels [50]. The ROS defense system is very important to reduce ROS levels in plants during abiotic stresses. With time, plants have evolved complex defense systems against the accumulation and production of ROS (Figure 2) [51].

## 6. Enzymatic Defense Systems 

Enzymatic defense systems against ROS include various enzymes such as glutathione reductases (GRs), dehydroascorbate reductases (DHARs), superoxide dismutases (SODs), monodehydroascorbate reductases (MDHARs), glutathione peroxidases (GPXs), catalases (CATs) and ascorbate peroxidases (APXs). The stresses associated with different defense mechanisms are listed in Table 1. Various enzymes involved in the defense system are discussed, such as SOD from the metallo-enzyme family of enzymes. The mechanism by which they decrease ROS includes the removal of O_2_^−^, converting it into O_2_ and H_2_O_2_ by a dismutation process that reduces the formation of OH^−^ [52]. In plants, SODs are present in chloroplasts (Cu/Zn-SOD), peroxisomes (Cu/Zn-SOD) and mitochondria (Mn-SOD). SOD in plants is increased by both biotic as well as abiotic stresses [52,53]. CAT are heme-containing enzymes that degrade two molecules of H_2_O_2_ into H_2_O and O_2_ by a dismutation process. Their tissue selectiveness depends on the place where they are present [54]. The expression of CAT is usually increased by drought, cold, heat and UV radiation [55,56]. Ascorbate-glutathione (AsA-GSH) has various reactions that enable the disposal of H_2_O_2_. This enzyme is mainly functional in chloroplasts, cytosol and mitochondria [57,58]. Plant glutathione-S-transferases (GSTs) are involved in programmed cell death and the response to biotic and abiotic stresses [59,60]. The enzymatic defense system against ROS is highly evolved as per the type of stress and place where there is oxidative damage.

### Non-Enzymatic Defense Systems 

Plants are armed with non-enzymatic and low molecular antioxidants such as carotenoids, α-tocopherol, GSH, AsA and proline. These are also involved in retrograde signaling [61,62]. GSH and AsA are the main elements that reduce H_2_O_2_ as they are quickly regenerated. The GSH-AsA cycle is responsible for the removal of ROS [63]. AsA acts as a cofactor as well as being responsible for the formation of tocopherol. GSH also plays an important role in ROS scavenging and as a raw material for various peroxidases. GSH can be located in mitochondria, nuclei, the endoplasmic reticulum and vacuoles [64]. Tolerance to high temperatures, drought and salinity is also enabled by the GSH content in plants [65,66]. Tocopherols are fat-hating compounds responsible for the disintegration of ROS; hence, they also protect membranes [67]. Carotenoids are lipid-soluble and mostly present in plastids. An enhanced concentration of these helps to build tolerance against various abiotic stresses [68]. Blue, red and purple pigmentation is provided by flavonoids in seeds, flowers and fruits. They are also reported to protect plants against various abiotic stresses such as high temperature and UV radiation [69,70,71,72].

**Table 1:** Different types of plants and their associated stresses and defense mechanisms.

Plant	Type of Stress	Defense System	Reference
*Triticum aestivum*	Drought	CAT and SOD activity increased	[73]
*Brassica napus*	Drought	Increased POD and CAT activity	[74]
*Vigna radiata*	Drought	Decreased ascorbate and increased DHA while decrease in their ratio	[75]
*Vigna radiata*	Salinity	Enhanced ascorbate and DHA activity	[76]
*Orysa sativa* L.	Salinity	Enhanced GSH and GB content, enhanced SOD activity	[66]
*Portulaca oleracea* L.	Elevated temperature	Increased SOD and POD activity	[77]
*Gossypium hirsutum*	Elevated temperature	Increased FeSOD and Cu/ZnSOD activity	[78]
*Triticum* spp.	Freezing temperature	Increased GST and APX activity	[79]
*Camellia sinensis* L.	Freezing temperature	Increased tea polyphenol to amino acid ratio	[80]
*Prunus persica* L. *Batsch*	Flooding	Increased CAT, POD and SOD activity	[81]
*Glycine max* L.	Heavy metal	Increased activity of both enzymes, i.e., SOD and POD	[82]
*Orysa sativa* L.	Heavy metal stress	Decreased ascorbate and DHA	[83]
*S. lycopersicum* L.	High light	SOD and POD activity decreased	[52]
*Malus crabapple*	High ozone	Enhanced POD, CAT and SOD	[74]
*Medicago sativa* L.	Alkalinity stress	Increased ascorbate, POD and CAT activities	[80]
*Triticum aestivum*	Acidic stress	Decreased ascorbate and GSH activity	[83]

## 7. Impact of ROS on Cell Biochemistry

**Figure 3:**
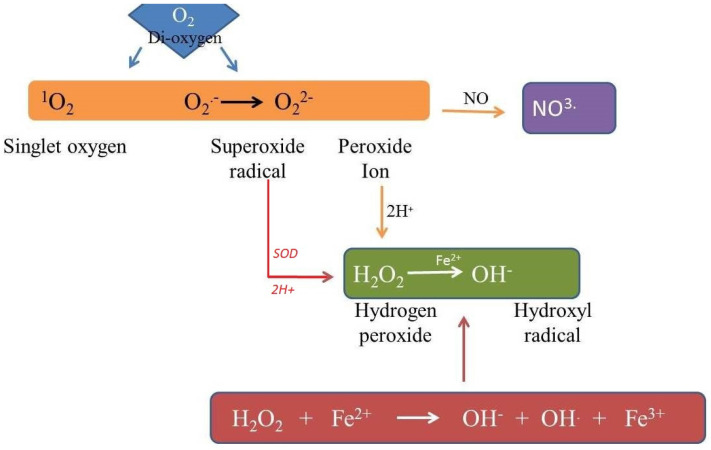
Atmospheric oxygen is shown to undergo excitation or reduction to form different ROS and reactive nitrogen species; super oxide dismutase is shown to form hydrogen peroxide (H_2_O_2_), which in turn reacts with Fe^2+^ to form hydroxyl radicals (OH^−^) via the Fenton reaction.

Reactive oxygen species are produced in the form of partially reduced or excited forms of atmospheric oxygen, e.g., singlet oxygen (^1^O_2_), hydrogen peroxide (H_2_O_2_) and hydroxyl radical (OH^.^) (Figure 3) [84]. They act as signaling molecules in cells but are also thought to be unavoidable toxic products of aerobic metabolism [19,85,86,87]. In cell signaling, the ROS molecules are highly versatile because of their diverse properties that include sites of production, different levels of reactivity and crossing potential to the biological membrane [1,61,88]. These molecules were first used by the cells to detect the harmful levels of atmospheric oxygen, or to regulate different metabolic activities, but they have evolved as potent signaling molecules to regulate almost all aspects of life in plants, animals and other eukaryotic organisms [24]. In the plant kingdom, these molecules regulate cell differentiation and development, stress signaling, plant interactions, systemic response, cell death and redox potentials [19,89,90,91,92]. The process of producing the ROS molecules occurs either by aerobic metabolism or by cellular antioxidative mechanisms constantly occurring in cells to prevent oxidative damage/stress [84]. Therefore, many antioxidant systems in the cell help in maintaining the ROS level at a non-toxic level, and any deviation from this balance could generate ROS-signaling reactions.

**Figure 4:**
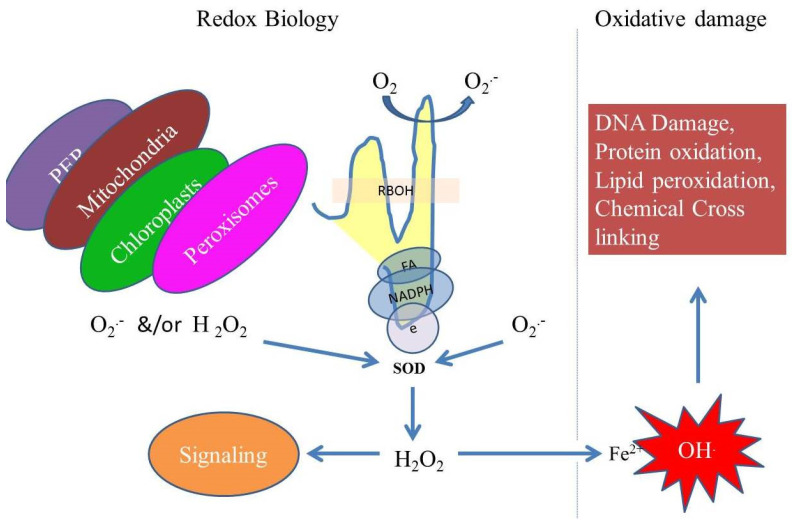
Integration of ROS and redox biology in cellular processes. Different cell organelles, including mitochondria, chloroplasts, peroxisomes and cell-wall-bound peroxidases (PER) and respiratory burst oxidase homologs (RBOHs), produce ROS that accumulate in the form of hydrogen peroxide (H_2_O_2_), resulting in the mediation of cell-to-cell signaling pathways. However, the presence of Fe^2+^ ions can cause cellular oxidative stress via hydroxyl radicals. These processes must be balanced and are crucial for redox biology for the regulation of the metabolism and other physiological and cellular functions.

Thus, ROS signaling is a highly regulated process and is mediated by the accumulation of ROS levels at specific cellular compartments. This may occur by plasma-membrane-bound NADPH-oxidase in plants and RBOH termed as NOX in animals. These are those enzymes that produce ROS in the apoplast [25,93,94,95]. This class of enzymes is also found in other cell organelles, such as endoplasmic reticulum (ER), vacuoles, nuclei, mitochondria or peroxisomes, and are mostly regulated via calcium and other phosphorylation/dephosphorylation reactions (Figure 4) [25,94]. In addition, ROS levels were mediated by peroxisomes at the apoplast as well as in the chloroplast, mitochondria and nuclei [1,24,89]. Therefore, a balance occurs in ROS production between the metabolically generated levels, the ROS diffusion rate and reactivity and the removal and perception in different cellular organelles, which on integration, generate a site-specific signaling system to regulate the antioxidant process and overall determine the cell-specific response to the stimulus [24]. It is important to remember the action of ROS, with the course of evolution in aerobic organisms, requires a specific solution at each cellular organelle to regulate ROS at each stage of their lifecycle. Hence, depending upon the nature of beneficial and toxic roles, they are named under a specific terminology called the “double-edged sword of life” [1,14,96,97]. Therefore, ROS are predominantly beneficial to cells, help in maintaining cellular and biochemical processes and regulate oxidative stress and cell death such as ferroptosis or necrosis [98,99]. 

## 8. Conclusions and Future Perspectives

The above discussion emphasizes ROS functioning as major determinants of cell fate as well as pivotal drivers of plant growth and development. Phytohormones such as abscisic acid and ethylene regulate stress-induced growth changes and control ROS production through antagonistic/synergistic interactions [32,33,34,80]. Components of the cell wall, such as the GASA/Snakin proteins, are also involved in the integration of ROS signaling and phytohormones, thus controlling growth and development processes along with pathogen resistance in plants. Insights into the regulation of these proteins by apoplastic ROS and their functions will provide a better understanding of the redox-dependent integration of extracellular and intracellular signals involved in plant growth and development. At present, there is currently a lack of knowledge about stress-inducible effects on redox post-translational modifications that help in the regulation of protein functions and protein localization [100]. The knowledge of PTMs is essential to completely understand the ROS-dependent mechanisms involved in environment-related plant growth and development. Research on redox-dependent programmed cell death demonstrates that ROS levels may increase above two different threshold levels altogether with an increase in ROS production that exceeds cellular scavenging capacity, both leading to cell death [32]. The current identification of plant genes that are involved in ROS-dependent PCD is solely the beginning of a new era during which excavating the surplus of gene regulatory networks controlling this mechanism will be momentous. Additionally, the screening of suppressors for ROS-induced cell death and cloning the remaining alleles has already been reported to regress or induce programmed cell death in Arabidopsis mutants. This will lead to an increase in the number of genes involved in the plant PCD pathways. Finalized transcriptome studies on genomes have led to the identification of hundreds of genes rapidly induced by ROS signaling [54]. Within this checklist, a group of novel genes involved in transduction and early ROS perception will undeniably be located. Functional screening of up-regulation or down-regulation of these genes and scoring them for their potential to regulate ROS-dependent cell death will help in their identification. Using this approach, along with the characterization of target proteins regulating post-translational modifications, such as nitrosylation and oxidation of proteins during cell death, will provide more depth into a comprehension of cell death in plants.

## Data Availability

The data presented in this study are available in review.

## Abbreviations

Reactive oxygen species (ROS), Singlet oxygen (^1^O_2_), Hydrogen peroxide (H_2_O_2_), Superoxide (O_2_^.−^), Hydroxyl radical (OH^.^), Photosystem (PS), Carbon dioxide (CO_2_), Ribulose-1,5-bisphosphate (RUBP), Nicotinamide adenine dinucleotide phosphate (NADPH), Catalase (CAT), Mitogen-activated protein kinase (MAPK), Microbe/pathogen associated molecular patterns (MAMPs/PAMPs), Resistance (R), Respiratory burst oxidase homolog (RBOH), Polyamine oxidase (PAO), NADPH Oxidases (NOX), Respiratory burst homolog D (RBOHD), RAC/ROP (Rho-GTPase in plants), Indole 3 butyric acid (IBR5), Abscisic acid (ABA), Jasmonic acid (JA), Salicylic acid (SA), Ascorbate peroxidases (APXs), Catalase (CAT), Superoxide dismutase (SOD), Glutathione peroxidases (GPX), Glutathione (GSH), Ascorbic acid (AA), Phenolic compounds and tocopherols (TOCs), Glutathione reductases (GRs), Dehydroascorbate reductases (DHARs), Monodehydroascorbate reductases (MDHARs), Ascorbate-glutathione (AsA-GSH), Gluthation-S-transferase (GSTs), Peroxidases (PER), Respiratory burst oxidase homologs (RBOHs), Gibberellic acid stimulated in Arabidopsis (GASA), Programmed cell death (PCD), Post-translational modifications (PTMs)

## References

[1] Huang H., Ullah F., Zhou D.X., Yi M., Zhao Y. (2019). Mechanisms of ROS regulation of plant development and stress responses. Front. Plant Sci..

[2] Hu H., Xiong L. (2014). Genetic Engineering and Breeding of Drought-Resistant Crops. Annu. Rev. Plant Biol..

[3] Walters D.R. (2003). Polyamines and plant disease. Phytochemistry.

[4] Jan B., Bhat T.A., Sheikh T.A., Wani O.A., Bhat M.A., Nazir A., Fayaz S., Mushtaq T., Farooq A., Wani S. (2020). Agronomic Bio-fortification of Rice and Maize with Iron and Zinc: A Review. Int. Res. J. Pure Appl. Chem..

[5] Purvis A.C. (1997). Role of the alternative oxidase in limiting superoxide production by plant mitochondria. Physiol. Plant..

[6] Zsigmond L., Rigó G., Szarka A., Szekely G., Otvos K., Darula Z., Medzihradszky K.F., Koncz C., Koncz Z., Szabados L. (2008). Arabidopsis PPR40 connects abiotic stress responses to mitochondrial electron transport. Plant Physiol..

[7] Schwarzländer M., Finkemeier I. (2013). Mitochondrial Energy and Redox Signaling in Plants. Antioxid. Redox Signal..

[8] Sako K., Futamura Y., Shimizu T., Matsui A., Hirano H., Kondoh Y., Muroi M., Aono H., Tanaka M., Honda K. (2020). Inhibition of mitochondrial complex I by the novel compound FSL0260 enhances high salinity-stress tolerance in Arabidopsis thaliana. Sci. Rep..

[9] Raja V., Majeed U., Kang H., Andrabi K.I., John R. (2017). Abiotic stress: Interplay between ROS, hormones and MAPKs. Environ. Exp. Bot..

[10] Gechev T.S., Van Breusegem F., Stone J.M., Denev I., Laloi C. (2006). Reactive oxygen species as signals that modulate plant stress responses and programmed cell death. BioEssays.

[11] Zeng J., Dong Z., Wu H., Tian Z., Zhao Z. (2017). Redox regulation of plant stem cell fate. EMBO J..

[12] Singh A., Kumar A., Yadav S., Singh I.K. (2019). Reactive oxygen species-mediated signaling during abiotic stress. Plant Gene.

[13] Gull R., Bhat T.A., Sheikh T.A., Wani O.A., Fayaz S., Nazir A., Saad A.A., Jan S., Nazir I., Nisa R. (2020). Climate change impact on pulse in India-A. J. Pharmacogn. Phytochem..

[14] Yamasaki H., Ogura M.P., Kingjoe K.A., Cohen M.F. (2019). d-cysteine-induced rapid root abscission in the water fern Azolla Pinnata: Implications for the linkage between d-amino acid and reactive sulfur species (RSS) in plant environmental responses. Antioxidants.

[15] Miller G., Suzuki N., Ciftci-Yilmaz S., Mittler R. (2010). Reactive oxygen species homeostasis and signalling during drought and salinity stresses. Plant Cell Environ..

[16] Joshi R., Wani S., Singh B., Bohra A., Dar Z., Lone A., Pareek A., Singla-Pareek S.L. (2016). Transcription Factors and Plants Response to Drought Stress: Current Understanding and Future Directions. Front. Plant Sci..

[17] Kimotho R.N., Baillo E., Zhang Z. (2019). Transcription factors involved in abiotic stress responses in Maize (*Zea mays* L.) and their roles in enhanced productivity in the post genomics era. PeerJ.

[18] Nadarajah K.K. (2020). ROS Homeostasis in Abiotic Stress Tolerance in Plants. Int. J. Mol. Sci..

[19] Goraya G.K., Asthir B. (2016). Magnificant role of intracellular reactive oxygen species production and its scavenging encompasses downstream processes. J. Plant Biol..

[20] Foyer C.H. (2018). Reactive oxygen species, oxidative signaling and the regulation of photosynthesis. Environ. Exp. Bot..

[21] Smirnoff N., Arnaud D. (2018). Hydrogen peroxide metabolism and functions in plants. New Phytol..

[22] Fichman Y., Mittler R. (2020). Rapid systemic signaling during abiotic and biotic stresses: Is the ROS wave master of all trades?. Plant J..

[23] Devireddy A.R., Arbogast J., Mittler R. (2020). Coordinated and rapid whole-plant systemic stomatal responses. New Phytol..

[24] McLachlan D.H. (2019). Systemic signalling, and the synchronization of stomatal response. New Phytol..

[25] Mittler R., Vanderauwera S., Suzuki N., Miller G., Tognetti V.B., Vandepoele K., Gollery M., Shulaev V., Van Breusegem F. (2011). ROS signaling: The new wave?. Trends Plant Sci..

[26] Suzuki N., Miller G., Morales J., Shulaev V., Torres M.A., Mittler R. (2011). Respiratory burst oxidases: The engines of ROS signaling. Curr. Opin. Plant Biol..

[27] Berriri S., Garcia A.V., Frey N.F.D., Rozhon W., Pateyron S., Leonhardt N., Montillet J.-L., Leung J., Hirt H., Colcombet J. (2012). Constitutively Active Mitogen-Activated Protein Kinase Versions Reveal Functions of Arabidopsis MPK4 in Pathogen Defense Signaling. Plant Cell.

[28] Singh G., Batra N., Salaria A., Wani O., Singh J. (2021). Groundwater quality assessment in Kapurthala district of central plain zone of Punjab using hydrochemical characteristics. J. Soil Water Conserv..

[29] Choudhury F.K., Rivero R.M., Blumwald E., Mittler R. (2017). Reactive oxygen species, abiotic stress and stress combination. Plant J..

[30] Peer W.A., Cheng Y., Murphy A.S. (2013). Evidence of oxidative attenuation of auxin signalling. J. Exp. Bot..

[31] Zwiewka M., Bielach A., Tamizhselvan P., Madhavan S., Ryad E.E., Tan S., Hrtyan M., Dobrev P., Vanková R., Friml J. (2019). Root adaptation to H_2_O_2_-induced oxidative stress by ARF-GEF BEN1-and cytoskeleton-mediated PIN2 trafficking. Plant Cell Physiol..

[32] Baba A.I., Rigó G., Ayaydin F., Rehman A.U., Andrási N., Zsigmond L., Valkai I., Urbancsok J., Vass I., Pasternak T. (2018). Functional Analysis of the Arabidopsis thaliana CDPK-Related Kinase Family: AtCRK1 Regulates Responses to Continuous Light. Int. J. Mol. Sci..

[33] Baba A.I., Andrási N., Valkai I., Gorcsa T., Koczka L., Darula Z., Medzihradszky K.F., Szabados L., Fehér A., Rigó G. (2019). AtCRK5 protein kinase exhibits a regulatory role in hypocotyl hook development during skotomorphogenesis. Int. J. Mol. Sci..

[34] Baba A.I., Valkai I., Labhane N.M., Koczka L., Andrási N., Klement É., Darula Z., Medzihradszky K.F., Szabados L., Fehér A. (2019). CRK5 protein kinase contributes to the progression of embryogenesis of Arabidopsis thaliana. Int. J. Mol. Sci..

[35] Cséplő Á., Zsigmond L., Andrási N., Baba A.I., Labhane N.M., Pető A., Kolbert Z., Kovács H.E., Steinbach G., Szabados L. (2021). The AtCRK5 Protein Kinase Is Required to Maintain the ROS NO Balance Affecting the PIN2-Mediated Root Gravitropic Response in Arabidopsis. Int. J. Mol. Sci..

[36] Xing Y., Chen W., Jia W., Zhang J. (2015). Mitogen-activated protein kinase kinase 5 (MKK5)-mediated signalling cascade regulates expression of iron superoxide dismutase gene in *Arabidopsis* under salinity stress. J. Exp. Bot..

[37] Jalmi S.K., Sinha A.K. (2015). ROS mediated MAPK signaling in abiotic and biotic stress-striking similarities and differences. Front. Plant Sci..

[38] Chhagan B.R., Sharma M.P., Sharma K.R., Samanta A., Owais A.W., Kachroo D., Kumar M., Razdan V.K., Sharma V., Mondal A.K. (2019). Impact of organic, inorganic and biofertilizers on crop yield and N, P and K uptake under rainfed maize-wheat cropping system. Int. J. Curr. Microbiol. Appl. Sci..

[39] Devireddy A.R., Zanadalinas S., Fichmen Y., Mittler R. (2021). Integration of reactive oxygen species and hormone signaling during abiotic stress. Plant J..

[40] Tanveer M., Ahmed H.A.I. (2020). ROS signalling in modulating salinity stress tolerance in plants. Salt and Drought Stress Tolerance in Plants.

[41] Shah A.N., Tanveer M., Abbas A., Fahad S., Baloch M.S., Ahmad M.I., Saud S., Song Y. (2021). Targeting salt stress coping mechanisms for stress tolerance in Brassica: A research perspective. Plant Physiol. Biochem..

[42] Bowler C., Camp W.V., Montagu M.V., Inze D., Asada P.K. (1994). Superoxide dismutase in plants. Crit. Rev. Plant Sci..

[43] Foyer C.H., Halliwell B. (1976). The presence of glutathione and glutathione reductase in chloroplasts: A proposed role in ascorbic acid metabolism. Planta.

[44] Hossain M., Asada K. (1987). Ascorbate-regenerating enzymes in chloroplasts. Indian J. Biochem. Biophys..

[45] Karpinska B., Karlsson M., Schinkel H., Streller S., Suss K.H., Melzer M., Wingsle G. (2001). A novel superoxide dismutase with a high isoelectric point in higher plants. Expression, regulation, and protein localization. Plant Physiol..

[46] Noctor G., Reichheld J.P., Foyer C.H. (2018). ROS-related redox regulation and signaling in plants. in Seminars in Cell & Developmental Biology. Semin. Cell Dev. Biol..

[47] Apel K., Hirt H. (2004). Reactive oxygen species: Metabolism, oxidative stress, and signal transduction. Annu. Rev. Plant Biol..

[48] Munné-Bosch S., Alegre L. (2004). Die and let live: Leaf senescence contributes to plant survival under drought stress. Funct. Plant Biol..

[49] Pang C.H., Wang B.S. (2008). Oxidative stress and salt tolerance in plants, in Progress in botany. Prog. Bot..

[50] Sharma P., Jha A.B., Dubey R.S. (2019). Oxidative stress and antioxidative defense system in plants growing under abiotic stresses. Handbook of Plant and Crop Stress.

[51] Berni R., Luyckx M., Xu X., Legay S., Sergeanty K., Hausman J.F., Lutts S., Cai G., Guirriero G. (2019). Reactive oxygen species and heavy metal stress in plants: Impact on the cell wall and secondary metabolism. Environ. Exp. Bot..

[52] Lu T., Meng Z., Zhang G., Qi M. (2017). Sub-high temperature and high light intensity induced irreversible inhibition on photosynthesis system of tomato plant (*Solanum lycopersicum* L.). Front. Plant Sci..

[53] Ahmad P., Jaleel C.A., Salem M.A., Nabi G., Sharma S. (2010). Roles of enzymatic and nonenzymatic antioxidants in plants during abiotic stress. Crit. Rev. Biotechnol..

[54] Mhamdi A., Chaouch S., Vanderauwera S., Vanbreusgem F., Noctor G. (2010). Catalase function in plants: A focus on Arabidopsis mutants as stress-mimic models. J. Exp. Bot..

[55] Caverzan A., Casassola A., Brammer S.P. (2016). Reactive oxygen species and antioxidant enzymes involved in plant tolerance to stress. Abiotic and Biotic Stress in Plants-Recent Advances and Future Perspectives.

[56] Sofo A., Scopa A., Nuzacci M., Vitti A. (2015). Ascorbate peroxidase and catalase activities and their genetic regulation in plants subjected to drought and salinity stresses. Int. J. Mol. Sci..

[57] Nishimura K., Sano M., Manami O., Nkauchi H., Yamaguchi T., Nakanishi M. (2011). Development of defective and persistent Sendai virus vector: A unique gene delivery/expression system ideal for cell reprogramming. J. Biol. Chem..

[58] Jimenez A., Hernandez J.A., Del Rio L.A. (1997). Evidence for the presence of the ascorbate-glutathione cycle in mitochondria and peroxisomes of pea leaves. Plant Physiol..

[59] Dixon D.P., Skipsey M., Edwards R. (2010). Roles for glutathione transferases in plant secondary metabolism. Phytochemistry.

[60] Gong H., Jiao Y., Hu W., Pua E.C. (2005). Expression of glutathione-S-transferase and its role in plant growth and development in vivo and shoot morphogenesis in vitro. Plant Mol. Biol..

[61] König K., Vaseghi M.J., Dreyer A., Dietz K.J. (2018). The significance of glutathione and ascorbate in modulating the retrograde high light response in Arabidopsis thaliana leaves. Physiol. Plant..

[62] Agati GAzzarello E., Pollastri S., Tattini M. (2012). Flavonoids as antioxidants in plants: Location and functional significance. Plant Sci..

[63] Zhang Y. (2013). Ascorbic Acid in Plants: Biosynthesis, Regulation and Enhancement.

[64] Koffler B.E., Bloem E., Zellnig G., Zechmann B. (2013). High resolution imaging of subcellular glutathione concentrations by quantitative immunoelectron microscopy in different leaf areas of Arabidopsis. Micron.

[65] Cheng M.C., Ko K., Chang W.L., Kuo W.C., Chen G.H., Lin T.P. (2015). Increased glutathione contributes to stress tolerance and global translational changes in Arabidopsis. Plant J..

[66] Hasanuzzaman MAlam M., Rahman A., Hasanuzzman M., Nhar K., Fujita M. (2014). Exogenous proline and glycine betaine mediated upregulation of antioxidant defense and glyoxalase systems provides better protection against salt-induced oxidative stress in two rice (*Oryza sativa* L.) varieties. BioMed Res. Int..

[67] Kamal-Eldin A., Appelqvist L.Å. (1996). The chemistry and antioxidant properties of tocopherols and tocotrienols. Lipids.

[68] Nisar N., Shan L.L., Lu S., Khin N.C., Pogson B.J. (2015). Carotenoid metabolism in plants. Mol. Plant.

[69] Petrussa E., Braidot E., Zancani M., Peresson C. (2013). Plant flavonoids—biosynthesis, transport and involvement in stress responses. Int. J. Mol. Sci..

[70] Winkel-Shirley B. (2002). Biosynthesis of flavonoids and effects of stress. Curr. Opin. Plant Biol..

[71] Das K., Roychoudhury A. (2014). Reactive oxygen species (ROS) and response of antioxidants as ROS-scavengers during environmental stress in plants. Front. Environ. Sci..

[72] Mansoor S., Kour N., Manhas S., Zahid S., Wani O.A., Sharma V., Wijaya L., Alyemeni M.N., Alsahi A.A., El-Serehy H.A. (2021). Biochar as a tool for effective management of drought and heavy metal toxicity. Chemosphere.

[73] Akram N.A., Shafiq F., Ashraf M. (2017). Ascorbic acid-a potential oxidant scavenger and its role in plant development and abiotic stress tolerance. Front. Plant Sci..

[74] Akram N., AIqbal M., Muhammad A., Ashraf M., Qurainy F.A., Shafiq S. (2018). Aminolevulinic acid and nitric oxide regulate oxidative defense and secondary metabolisms in canola (*Brassica napus* L.) under drought stress. Protoplasma.

[75] Nahar K., Hasanuzzaman M., Alam M., Rahman A., Mahmud J., Suzuki T., Fuji M. (2017). Insights into spermine-induced combined high temperature and drought tolerance in mung bean: Osmoregulation and roles of antioxidant and glyoxalase system. Protoplasma.

[76] Nahar K., Hasanuzzaman M., Alam M., Rahman A., Mahmud J., Suzuki T., Fuji M. (2016). Polyamines confer salt tolerance in mung bean (*Vigna radiata* L.) by reducing sodium uptake, improving nutrient homeostasis, antioxidant defense, and methylglyoxal detoxification systems. Front. Plant Sci..

[77] Jin R., Wang Y., Liu R., Gou J., Chan Z. (2016). Physiological and metabolic changes of purslane (*Portulaca oleracea* L.) in response to drought, heat, and combined stresses. Front. Plant Sci..

[78] Sekmen A.H., Ozgur R., Uzilday B., Turkman I. (2014). Reactive oxygen species scavenging capacities of cotton (*Gossypium hirsutum*) cultivars under combined drought and heat induced oxidative stress. Environ. Exp. Bot..

[79] Majláth I., Eva C., Tajti J., Khalil R., Elsayed N., Darko E., Szalai G., Janda T. (2020). Exogenous methylglyoxal enhances the reactive aldehyde detoxification capability and frost-hardiness of wheat. Plant Physiol. Biochem..

[80] Li Q., Wang W., Wang W., Zhangh G., Liu Y., Wang Y., Wang W. (2018). Wheat F-box protein gene TaFBA1 is involved in plant tolerance to heat stress. Front. Plant Sci..

[81] Malerba M., Cerana R. (2018). Effect of selenium on the responses induced by heat stress in plant cell cultures. Plants.

[82] Bhat J.A., Faizan M., Bhat M.A., Huang F., Yu D., Ahmad A., Bajguz A., Ahmad P. (2022). Defense interplay of the zinc-oxide nanoparticles and melatonin in alleviating the arsenic stress in soybean (*Glycine max* L.). Chemosphere.

[83] Bhuyan M., Parvin K., Mohsin S.M., Mahmud J.A., Hassanuzzman M., Fujita M. (2020). Modulation of cadmium tolerance in rice: Insight into vanillic acid-induced upregulation of antioxidant defense and glyoxalase systems. Plants.

[84] Halliwell B., Gutteridge J.M. (2015). Free Radicals in Biology and Medicine.

[85] König J., Muthuramalingam M., Dietz K.J. (2012). Mechanisms and dynamics in the thiol/disulfide redox regulatory network: Transmitters, sensors and targets. Curr. Opin. Plant Biol..

[86] Mignolet-Spruyt L., Xu E., Idänheimo N., Hoeberichts F.A., Mühlenbock P., Brosché M., Van Breusegem F., Kangasjärvi J. (2016). Spreading the news: Subcellular and organellar reactive oxygen species production and signalling. J. Exp. Bot..

[87] Vaahtera L., Brosché M., Wrzaczek M., Kangasjärvi J. (2014). Specificity in ROS signaling and transcript signatures. Antioxid. Redox Signal..

[88] Foyer C.H., Noctor G. (2013). Redox Signaling in Plants.

[89] Mittler R., Vanderauwera S., Gollery M., Van Breusegem F. (2004). Reactive oxygen gene network of plants. Trends Plant Sci..

[90] Hussain N., Bahar F.A., Mehdi S.S., Bhat M.A., Hussain A., Kanth R.H., Sheikh T., Ahmad R., Wani O.A., Nazim H.M. (2021). A Brief Insight into Nutritional Deficiencies in Pulses and their Possible Management Strategies A Review. Curr. J. Appl. Sci. Technol..

[91] Mahdi S.S., Jan R., Jehangir I.A., Hussain A., Bhat M.A., Dhekale B., Ahmed L., Sofi N.R., Bangroo S.A., Qureshi A.M. (2021). Farmer’s perception of climate change and adaptation strategies under temperate environmental conditions of Kashmir, India. J. Agrometeorol..

[92] Sumimoto H. (2008). Structure, regulation and evolution of Nox-family NADPH oxidases that produce reactive oxygen species. FEBS J..

[93] Laurindo F.R., Araujo T.L., Abrahao T.B. (2014). Nox NADPH oxidases and the endoplasmic reticulum. Antioxid. Redox Signal..

[94] Sirokmány G.Á., Geiszt M. (2016). Nox/Duox family of NADPH oxidases: Lessons from knockout mouse models. Trends Pharmacol. Sci..

[95] Boyd E.S., Thomas K.M., Dai Y., Boyd J.M., Outten F.W. (2014). Interplay between oxygen and Fe–S cluster biogenesis: Insights from the Suf pathway. Biochemistry.

[96] Zazai K.G., Wani O.A., Ali A., Devi M. (2018). Phytoremediation and carbon sequestration potential of agroforestry systems: A review. Int. J. Curr. Microbiol. App. Sci..

[97] Xie Y., Hou W., Song X., Yu Y., Huang J., Sun X., Kang R., Tang D. (2016). Ferroptosis: Process and function. Cell Death Differ..

[98] Conrad M., Angeli JP F., Vandenabeele P., Stockwell B.R. (2016). Regulated necrosis: Disease relevance and therapeutic opportunities. Nat. Rev. Drug Discov..

[99] Nakashima K., Yamaguchi-Shinozaki K. (2013). ABA signaling in stress-response and seed development. Plant Cell Rep..

[100] Choudhary A., Kumar A., Kaur N. (2020). ROS and oxidative burst: Roots in plant development. Plant Divers..

